# Development of a Zebrafish S1500+ Sentinel Gene Set for High-Throughput Transcriptomics

**DOI:** 10.1089/zeb.2018.1720

**Published:** 2019-08-01

**Authors:** Michele R. Balik-Meisner, Deepak Mav, Dhiral P. Phadke, Logan J. Everett, Ruchir R. Shah, Tamara Tal, Peter J. Shepard, B. Alex Merrick, Richard S. Paules

**Affiliations:** ^1^ Sciome, LLC, Research Triangle Park, North Carolina.; ^2^ National Health and Environmental Effects Research Laboratory, Office of Research and Development, U.S. Environmental Protection Agency, Research Triangle Park, North Carolina.; ^3^ BioSpyder Technologies, Incorporated, Carlsbad, California.; ^4^ Division of the National Toxicology Program, National Institute of Environmental Health Sciences, National Institutes of Health, Research Triangle Park, North Carolina.

**Keywords:** zebrafish, S1500, S1500+, transcriptomics

## Abstract

Sentinel gene sets have been developed with the purpose of maximizing the information from targeted transcriptomic platforms. We recently described the development of an S1500+ sentinel gene set, which was built for the human transcriptome, utilizing a data- and knowledge-driven hybrid approach to select a small subset of genes that optimally capture transcriptional diversity, correlation with other genes based on large-scale expression profiling, and known pathway annotation within the human genome. While this detailed bioinformatics approach for gene selection can in principle be applied to other species, the reliability of the resulting gene set depends on availability of a large body of transcriptomics data. For the model organism zebrafish, we aimed to create a similar sentinel gene set (Zf S1500+ gene set); however, there is insufficient standardized expression data in the public domain to train the gene correlation model. Therefore, our strategy was to use human-zebrafish ortholog mapping of the human S1500+ genes and nominations from experts in the zebrafish scientific community. In this study, we present the bioinformatics curation and refinement process to produce the final Zf S1500+ gene set, explore whole transcriptome extrapolation using this gene set, and assess pathway-level inference. This gene set will add value to targeted high-throughput transcriptomics in zebrafish for toxicogenomic screening and other research domains.

## Introduction

Tox21 is a multiagency U.S. government program^1,2^ with the objective to improve chemical hazard assessment through approaches that can obtain high-quality data in an efficient manner that complements traditional animal studies, moving toward a systems biology approach to toxicology. ToxCast, an additional effort from the U.S. EPA, was also instituted to predict toxicity and prioritize thousands of chemicals with limited previous hazard information.^3^ *In vitro* high-throughput assays have been paramount to achieve these goals. High-throughput transcriptomic (HTT) approaches enable toxicogenomic screening that can provide dose–response information on gene expression alterations, which might be useful in risk assessment determinations. 

To produce transcriptomic data on a large number of samples at a rapid pace, targeted assays have been developed that allow a much greater degree of multiplexing compared to traditional RNA-Seq methods.^4^ The human S1500+ gene set was created as a transcriptomic subset representative of biologically diverse genes, and was developed in a data-driven manner.^5,6^ It can be utilized to accurately predict human pathway perturbations and biological relationships.^7^ Co-expression patterns from large repositories of microarray and RNA-Seq data can be used in conjunction with this gene set to extrapolate expression for the full nonmeasured transcriptome.^6^

Two notable follow-up steps have also been performed since creating the human S1500+ gene set. (1) A TempO-Seq (BioSpyder Technologies, Inc., Carlsbad, CA) platform has been created to measure gene expression of the human S1500+ genes.^4^ This platform allows researchers to directly measure the S1500+ genes without having to design their own probes. (2) After creating the human S1500+ gene set, the same methods were employed to select gene sets for mouse and rat for use in animal toxicology studies (https://ntp.niehs.nih.gov/results/tox21/s1500-gene-set-consensus-strategy-index.html), and performance of the gene sets as well as accuracy of the TempO-Seq platforms have been assessed.^8^

Each gene set is representative of full transcriptomic space in the target species. Accurate assessments of biological relationships can be made from the measured genes alone or expression for the full transcriptome can be extrapolated before making inference. Both the creation of an HTT platform and the inclusion of other model organism species facilitate the utility of the S1500+ approach and immediate use of the gene sets.

Zebrafish (*Danio rerio*) is an important model organism in vertebrate genomics and toxicity testing. They are a particularly useful model for early vertebrate development. Their high genetic homology with humans (∼70%), rapid, *ex vivo*, and well-characterized development,^9,10^ highlights their utility for chemical screens. For example, the zebrafish model has been used to address Tox21 aims to prioritize ToxCast chemicals in medium-to-high-throughput screens.^11,12^

Beyond chemical prioritization and safety assessment, approaches stemming from ToxCast chemical prioritization efforts have advanced toward gaining genetic insight into differential susceptibility to specific chemical exposures.^13^ Zebrafish transcriptomics has also gained traction in toxicogenomic studies where differential gene expression was assessed after chemical or metal exposures.^14–16^ As an *in vivo* model with many emerging applications, the zebrafish is a compelling option for building the next species-specific S1500+ gene set.

A major challenge in creating a zebrafish S1500+ gene set is the lack of sufficient zebrafish gene expression data in public repositories that would be required for the *de novo* gene selection approach used in the creation of the human S1500+ gene set.^6^ Therefore, we developed an alternative gene selection approach that identifies zebrafish orthologs of the existing human S1500+ gene set as a proxy for a diverse and biologically meaningful subset of the zebrafish transcriptome. In addition, biologically and transcriptionally relevant zebrafish genes were recommended by experts for inclusion in the gene set. The addition of expert-curated zebrafish genes ensures the utility of the gene set for making inferences for vertebrate-specific toxicogenomics, in addition to covering orthologous mammalian transcriptomic space.

Another significant challenge in creating a zebrafish S1500+ gene set relates to the multiple genome duplication events in zebrafish evolution that gave rise to multiple zebrafish paralogs linked to a single human orthologous gene.^17^ In this study, we present our methods for determining zebrafish orthologs of human S1500+ genes based on multiple ortholog databases, selecting a single zebrafish ortholog from duplicated genes, and building the final zebrafish Zf S1500+ gene set in conjunction with gene nominations from developmental neurotoxicity (DNT) and zebrafish field experts.

After building the Zf S1500+ gene set, we demonstrate the feasibility of extrapolating the full zebrafish transcriptome from this subset to make pathway-level inferences using the human canonical pathways in the Molecular Signature Database (MSigDB).^18^ Finally, we evaluate the utility of the gene set and full transcriptome extrapolation using Kyoto Encyclopedia of Genes and Genomes^19^ (KEGG) pathway and Gene Ontology^20,21^ (GO) enrichment (from this gene set alone and from the results of two extrapolation approaches) using real zebrafish gene expression data.

## Materials and Methods

### Assessing homology with human S1500+

The human S1500+ TempO-Seq^4^ platform contains 2,711 unique genes that were previously selected to optimize representation of the diverse biology of the human whole transcriptome (WTr).^6^ To ensure that the zebrafish platform would adequately cover the same biological space, we identified zebrafish orthologs of human S1500+ genes based on multiple sources. Our method for determining human-zebrafish orthologous gene pairs was as follows:

#### Compilation of ortholog databases

It has been estimated that human and zebrafish diverged from a common ancestor ∼450 million years ago,^22^ and the evolutionary distance between the human and zebrafish genomic sequences are 1.8 neutral substitutions per site compared to 0.5–2.0 substitutions per site for human-to-mouse and zebrafish-to-mouse, respectively.^23^ In addition, zebrafish underwent multiple genome duplication events,^17^ resulting in duplicated genes with loss of function, neofunctionalization, subfunctionalization, or altered spatiotemporal gene expression. Therefore, defining orthologs genome wide between these species is not straightforward.

There are multiple methods for mapping orthologs by sequence homology in the literature.^24^ To perform a thorough analysis, three ortholog databases were used to determine human and zebrafish orthologs. Table 1 shows the most recent genome builds and file updates for each database.

**Table 1.** Release and Genome Build History for the Three Ortholog Databases

	*NCBI HomoloGene*	*ZFIN*	*Ensembl*
Zebrafish genome build	Zv9	GRCz11	GRCz11
Build date	July 15, 2010	May 9, 2017	May 9, 2017
Human genome build	GRCh38	GRCh38	GRCh38
Build date	December 17, 2013	Patch release 12 from December 21, 2017	Patch release 12 from December 21, 2017
Date of last ortholog file update before download	May 2014	July 2018	July 2018

ZFIN, Zebrafish Information Network.

##### NCBI HomoloGene

We downloaded HomoloGene build 68 (released April 2014).^25^ This is a widely used database in the field,^26–29^ but has not been recently updated and uses a zebrafish genome build from 2010. Hence, in addition to this resource, we looked for other ortholog databases that use the latest genome builds and are updated more regularly.

##### Zebrafish Information Network human ortholog file

The Zebrafish Information Network (ZFIN) orthologs were downloaded from https://zfin.org/downloads/human_orthos.txt on July 16, 2018 (curation date July 15, 2018).^30,31^ This file is updated daily using the current genome builds. ZFIN ortholog mapping is based on a combination of experimental evidence (including amino acid sequence comparison, coincident expression, synteny, functional complementation, nucleotide sequence comparison, and phylogenetic trees) and manual curation.

##### Ensembl orthologs

Ensembl orthologs were downloaded from http://useast.ensembl.org/biomart/martview (using Ensembl Genes 93 database, zebrafish genome GRCz11, and filtering for genes with orthologs in human genome GRCh38.p12) on July 25, 2018.^32^ The data in the file were curated by Ensembl in July 2018 with the release of Ensembl 93 and is based on the most recent genome builds. The Ensembl ortholog methodology^33,34^ builds protein trees and applies multiple genome alignment to use gene trees to represent the evolutionary history of gene families, which evolved from a common ancestor. Ortholog mappings are labeled as “high confidence” if they meet the following thresholds: a minimum sequence identity of 25% (in both directions), minimum gene order conservation (GOC) score of 50, and minimum whole genome alignment score of 50. We only included orthologs that were marked as “high confidence” to limit false positives.

#### Combination of ortholog databases

After download, each database was curated to only include genes with current Entrez IDs (based on the NCBI GENE_INFO tables for zebrafish and human) and Ensembl IDs (in Ensembl annotation 93, released July 2018). We combined the ortholog pairs across all three sources to construct one unified mapping, the Combined Zebrafish Ortholog Database (CZfO db) (Supplementary Data S1). Entrez IDs were used as the unique identifiers in each species to match up all genes across all datasets used in the analysis.

Every gene on the human S1500+ platform was assessed using the combined set of orthology mappings based on all three databases outlined above (CZfO db). Overall, a gene was considered to have an ortholog in the other species if one or more ortholog pairs were present in at least one of the three databases above.

### Expert-curated list (G_F_) and manually curated list (G_M_)

Assurance that the final Zf S1500+ list would be relevant to the zebrafish research community made it necessary to include zebrafish genes with known biological roles, in addition to sequence-based orthologs of the human S1500+ genes. Collaborators in multiple fields nominated additional genes of biological interest in zebrafish genetics and/or toxicology research (Supplementary Data S2). These included genes involved in specific biological functions (Table 2) (e.g., DNT, central nervous system development, blood–brain barrier, and serotonin biosynthesis). We refer to these 870 nominations as G_F_ (genes nominated for specific biological functions). In addition, a starting list of orthologs was provided by BioSpyder Technologies, Inc. These zebrafish orthologs were previously obtained for the human S1500+ genes using the Ensembl Compara^35^ method and downloaded from http://useast.ensembl.org/biomart/martview for Ensembl 84. We refer to this list of 2,943 genes as G_M_ (manually curated human S1500+ homologs) (Supplementary Data S2). One hundred and one genes were provided on both G_F_ and G_M_.

**Table 2.** Biological Categories of Expert-Nominated Genes in G_F_

*Category*
Blood–brain barrier
Brain development
Cardiovascular function
Circadian rhythm
CNS development
Dioxin response pathway
DNT
Estrogen signaling
Gonad development
Gut development
HV
HV-brain
HV-circadian
Kidney development/function
Lateral line
Myelination
Neuronal migration
Pancreas development
Serotonin biosynthesis
Thyroid

CNS, central nervous system; DNT, developmental neurotoxicity; HV, ventral zone of hypothalamus.

The G_F_ and G_M_ lists consisted of a combination of gene symbols (names) and gene Ensembl IDs (Supplementary Data S2). We matched gene symbols (or Ensembl IDs when the symbol had no match) from this list to Entrez IDs using the zebrafish GENE_INFO table. This was done so that the genes could be appropriately mapped to our compiled ortholog database (CZfO db), which used Entrez IDs as the unique gene identifier. The final usage of the expert-curated list (G_F_ and G_M_) is explained in detail in the procedure, in the next section.

### Building the Zf S1500+ gene set

Due to multiple genome duplication events in teleost fish evolutionary history,^36^ and, in part, to the large evolutionary divergence between human and zebrafish, many human genes have multiple orthologs in zebrafish.^9^ As a result, the inclusion of all zebrafish orthologs of each human S1500+ gene would substantially increase the size of the Zf S1500+ platform and contains many genes that are redundant with respect to human biology, thereby reducing the cost-effectiveness of the platform for HTT toxicogenomic studies. We therefore built the Zf S1500+ gene set to contain a comparable number of genes to the human S1500+ platform, while maximizing coverage of the same biological functions and pathways. This involved filtering redundant orthologs, while preferentially choosing important evolutionarily distinct zebrafish gene copies.

A caveat to this approach is that if duplicated genes exhibit neofunctionalization, subfunctionalization, or altered spatiotemporal gene expression patterning, toxicogenomic gene expression profiling using this gene set may miss potentially important chemical-dependent gene expression changes in duplicated genes with new or different functions or those that are expressed at potentially more sensitive developmental windows.

**FIG. 1.**
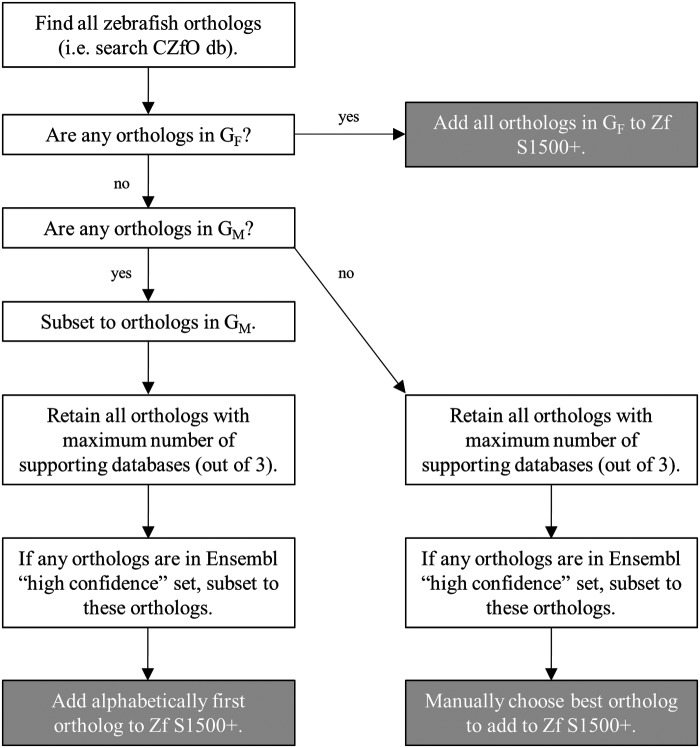
Creation of Zf S1500+: diagram describes selection process for the zebrafish orthologs of each human S1500+ gene for inclusion on the Zf S1500+ gene set. The workflow proceeds until there is only one ortholog to add to the list, unless otherwise stated. If at any step only one gene remains, then it is selected for inclusion, and the next iteration of workflow for the next human gene begins.

The procedure to choose ortholog(s) for one human S1500+ gene to add to the Zf S1500+ gene set is outlined here and displayed in Figure 1. This procedure was followed until a single best ortholog was identified, or if a subset of multiple orthologs was added to Zf S1500+ in step 2 (to retain all expert-nominated genes G_F_).

(1) All zebrafish orthologs for the human S1500+ gene were determined using CZfO db. A human gene was considered to have a zebrafish ortholog if an ortholog was present in at least one of the three ortholog resources.
(2) If any zebrafish ortholog(s) was present on the expert-curated list with an assigned biological function (genes in G_F_), then those zebrafish orthologs were all chosen, and the remaining steps were skipped. This was done to ensure that all genes nominated for specific biological functions were included in the final Zf S1500+ gene set.
(3) The set of possible orthologs for the human gene was then compared to the manually curated list (G_M_). If any of the orthologs was in G_M_, then only the subset of G_M_ genes was considered. This was done to preferentially choose from the curated set of orthologs if any existed. Otherwise, all orthologs were used for further processing.
(4) The set of possible orthologs was then reduced based on the strength of evidence of each orthologous relationship. Evidence strength was first assessed based on the number of databases that reported the ortholog relationship. The orthologs supported by the most databases were retained for further processing.
(5) For cases with multiple orthologs supported by an equal number of databases, ortholog pairs supported by the Ensembl high-confidence dataset were considered to have stronger evidence than other ortholog pairs because this mapping had the most stringent inclusion criteria of the three databases used and was based on the most recent build of the zebrafish genome (GRCz11). We therefore subset to Ensembl high-confidence orthologs if any existed.
(6) If the remaining orthologs came from G_M_, then the alphabetically first ortholog was chosen for Zf S1500+. For example, for the human gene ACOT9, zebrafish ortholog acot9.1 was retained over acot9.2. This was performed as a consistent, reproducible approach to choose the final gene when we had reason to believe that they were all transcriptionally relevant (due to presence on the list of curated human S1500+ orthologs).
(7) If the remaining orthologs were not from the expert-curated list, then we manually curated the NCBI gene entries to select the ortholog to add to Zf S1500+. The gene with the highest validation confidence (validated > provisional > predicted), the most related articles in PubMed, and the most pathway and/or functional annotations were chosen. These choices were made to ensure evidence was present in the literature for the transcriptional activity of the chosen gene. This process was performed for 17 genes (Supplementary Data S3).

Steps 1–7 were repeated for each of the 2,711 human S1500+ genes. All remaining zebrafish genes from G_F_ were then added to form the complete Zf S1500+ gene set.

### WTr and pathway coverage

The impact of making inference using Zf S1500+ was assessed by determining which genes and pathways would result from use of this targeted gene set relative to pathway-level identification using whole-genome sequencing results.

The human full transcriptome was curated using all extrapolated^6^ (using large collection of publicly available RNA-Seq data^37^ to train the model) and measured human S1500+ genes. We defined the full (full = measured+extrapolated) zebrafish transcriptome as the combination of (1) all zebrafish genes in the final Zf S1500+ gene set and (2) all other zebrafish genes from CZfO db for which a human ortholog exists in the full human (measured in and extrapolated from human S1500+) transcriptome. Specifically, this is the combination of the measured genes when using the Zf S1500+ platform and the genes that can be extrapolated after converting to the human transcriptomic space. The CZfO db was used to assess homology between zebrafish and human at the whole human transcriptome level.

Potential biological shortcomings in using Zf S1500+ to inform human pathway analysis were considered by examining pathway coverage based on the human S1500+, Zf S1500+, and human/zebrafish full (measured+extrapolated) transcriptomes. Canonical pathways (C2-CP) from the MSigDB (v6.2) database^18,38,39^ were downloaded from the Broad Institute. Gene coverage was assessed for each of the C2-CP and Hallmark sets in MsigDB.

### Assessment of Zf S1500+ using published RNA-Seq transcriptome gene expression data

#### Assessment of the Zf S1500+ genes for making accurate pathway-level conclusions

Four publicly available RNA-Seq datasets from Gene Expression Omnibus^40^ (GEO) were selected:

- BPA dataset (GSE113676)^41^: 12 samples of zebrafish embryos exposed to varying concentrations of bisphenol A (3 replicates each for control and 3 exposure concentrations)
- Caudal fin dataset (GSE76564)^42^: 4 samples (2 regenerating after amputation and 2 uninjured), each a pool of 10 fins
- Ablated heart dataset (GSE75894)^42^: 4 samples (2 regenerating following genetic ablation of cardiomyocytes and 2 uninjured), each a pool of 10 hearts
- Eye dataset (GSE100062)^43^: 8 samples (4 GFP+ fluorescence-activated cell sorting (FACS)-sorted rods and 4 GFP− FACS-sorted nonrods from retina); each sample contained the 2 retinas from each of 4 fish

Gene-level count data were downloaded from the Supplementary Data for each series. Ensembl IDs were converted to Entrez IDs, expression was normalized using simple transcripts per million normalization and log2 transformed, differential analysis was performed using *t*-tests, fold change and *p*-value cutoffs were used to determine differentially expressed genes (DEGs), and DAVID^44,45^ 6.8 was used to determine differentially enriched GO (BP_DIRECT, CC_DIRECT, and MF_DIRECT) and KEGG pathways (DEPs = Differentially Enriched Pathways). DEGs were determined to be genes with absolute value of fold change ≥1.5 and distributional *p*-value ≤0.01. DEPs required a minimum of three genes per pathway on the DEG list and *p*-value ≤0.05.

The caudal fin dataset had stronger gene expression signal; therefore, we strengthened the *p*-value thresholds to 0.001 for DEG analysis for this dataset to focus on a smaller subset of DEGs with stronger statistical evidence, for use in the downstream pathway analysis for this dataset. For the ablated heart dataset, we used the more stringent DEP *p*-value cutoff of 0.01 to focus on the biological conclusions coming from a smaller set of most relevant pathways. The fold change cutoffs remained the same for all studies to ensure that all statistically significant genes are biologically relevant (i.e., have meaningful fold change values between the study groups).

This analysis was performed on all genes (WTr) in each series. For an initial assessment of Zf S1500+ coverage of DEGs, we simply looked at how many DEGs determined in a given study were genes in the Zf S1500+ gene set. This value was then compared to DEG coverage of three randomly generated sets of genes of the exact same size as Zf S1500+ (3,055 genes with both Entrez and Ensembl IDs). The same three sets of random genes (Random1, Random2, and Random3) were used for each of the four series.

In addition, the full DEG and DEP analyses were performed on Zf S1500+ and all three Random datasets. For a given gene set, this was done by removing RNA-Seq genes not in that gene set from the count data, performing normalization using only the subset of measured transcriptome, and completing the whole process outlined above. DEPs obtained from each of these gene sets were compared to each other and to the DEPs identified in the WTr analysis.

Since overlap of DEPs alone may not relay when two DEP lists lead to similar biological conclusions (when similar, but not identical pathways are significant in different lists), we also looked at the percent of Keywords from the WTr RNA-Seq analysis that appeared in the name of any pathway in the specified set. Keywords were determined as any one- or two-word sequence that appeared in the name of at least two WTr DEPs. Common words that did not describe useful biology (e.g., “pathway” or “complex”) were not included as Keywords. The Keywords, frequencies, and set inclusion can be found in the Supplementary Data for each series comparison.

#### Assessment and comparison of two extrapolation methods

Two methods of extrapolation were tested:

(a) “Human Extrapolation”: Extrapolation using human training data (approach described in Materials and Methods “WTr and Pathway Coverage” and “WTr Extrapolation from Zf S1500+” sections).
(b) “Zebrafish Extrapolation”: Extrapolation using zebrafish training data.

For extrapolation in the human transcriptomic space [for (a) above], methods from our recent publication^6^ were used. This extrapolation approach incorporates use of principal component regression^46^ and has been updated to use a quality-filtered subset (reduced from 125,501 to 64,514 samples) of a large collection of publicly available RNA-Seq data^37^ to train the model.

Extrapolation in the zebrafish transcriptomic space [for (b) above] was performed using the same principal component regression methods on quality-filtered (reduced from 4,004 to 1,791 samples) zebrafish RNA-Seq data (https://amp.pharm.mssm.edu/archs4/archs4zoo.html). Filtering criteria involved requiring samples to meet the following minimum thresholds: 1,000,000 aligned reads, nonzero expression for 35% of WTr genes, and nonzero expression for 35% of species-specific S1500+ genes. Samples were also required to have 90% of the total reads mapped to at least 1,000 genes.

For each of the four GEO series described in the previous section, normalized gene counts from the Zf S1500+ genes were used exclusively, and both extrapolation approaches were applied. Following extrapolation, the same methods discussed previously were used to determine DEGs and DEPs separately for each approach and the results were compared to each other and to the WTr results (true results had all genes been measured). To limit any bias in extrapolation results, we made sure that none of the samples in any of these three GEO series was part of the zebrafish training set for approach (b) above [approach (a) does not include any zebrafish training data].

## Results

### Assessment of ortholog databases

Our logic for creating the Zf S1500+ gene set was to cover the same transcriptomic space as human S1500+ through the use of zebrafish orthologs, despite genome duplication events that often resulted in multiple copies of each gene. This first involved compiling a master zebrafish-human ortholog database by curating existing resources (CZfO db) (Supplementary Data S1).

**FIG. 2.**
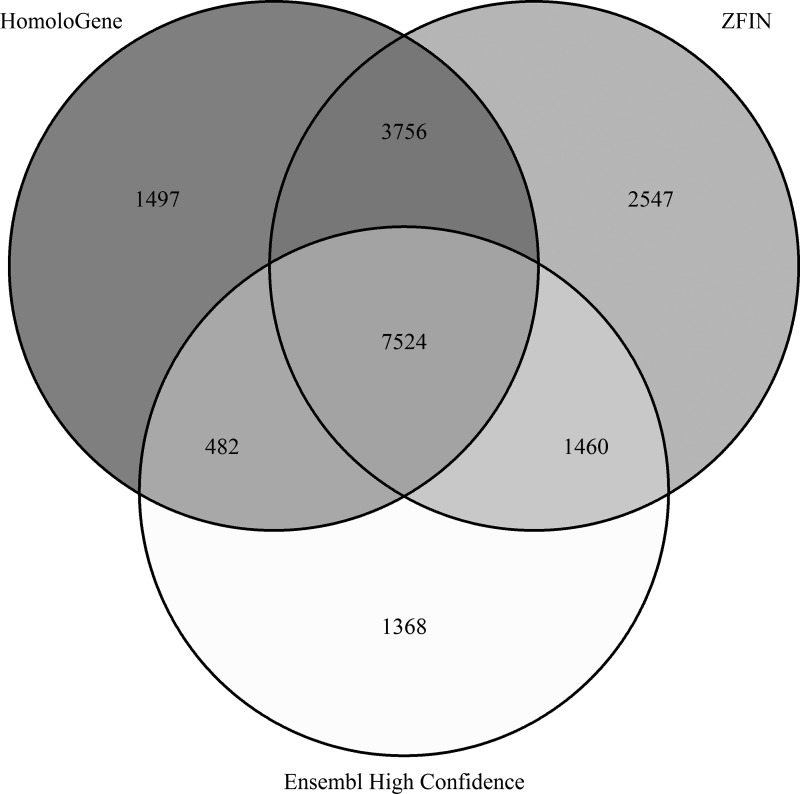
Venn Diagram of human-zebrafish ortholog gene pairs present in each of three databases (HomoloGene, ZFIN, and Ensembl “high confidence”) after filtering out genes with no current NCBI Entrez ID or Ensembl ID. A gene pair designates an orthologous relationship between one human and one zebrafish gene; therefore, genes with one-to-many or many-to-one orthologous relationships are counted for each unique pairing. ZFIN, Zebrafish Information Network.

After processing and integrating three ortholog databases (i.e., HomoloGene, ZFIN, and Ensembl “high confidence”), we assessed the similarity across each source. The three ortholog databases each had distinct sets of orthologs (one-to-one, one-to-many, and many-to-one), but the majority of the orthologous pairs are common among ≥2 databases (Fig. 2). The ZFIN dataset is the largest compilation of orthologs. Ensembl “high confidence” is the smallest, since it is the most restrictive. As a result, we determined that the strategy of assigning the highest strength of evidence to ortholog pairs present in this dataset was appropriate. Overall, we determined the combination of these datasets (CZfO db) (Supplementary Data S1) to be a comprehensive set of known and predicted orthology between zebrafish and human.

### Creation of the Zf S1500+ gene set

The ortholog mapping was then used to build Zf S1500+ to mirror the biological diversity of human S1500+. The current BioSpyder Technologies, Inc. human S1500+ platform has 2,711 unique human genes. Of these, there were 428 (16%) human S1500+ genes for which there are no zebrafish orthologs in CZfO db (Table 3 and Supplementary Data S3). These genes could not become part of the Zf S1500+ platform. Around 2,283 (84%) human genes were found to have at least one zebrafish ortholog in CZfO db (Table 3). The Zf S1500+ gene set was built to include at least one ortholog for all of these human genes, in addition to expert-nominated zebrafish genes with important biological functions.

**Table 3.** Zebrafish Orthologs for Human S1500+ Genes

*Human S1500+*	*Human S1500+ genes with zebrafish orthologs*				*Human S1500+ genes with no zebrafish orthologs*
	*NCBI HomoloGene*	*ZFIN*	*Ensembl*	*HomoloGene, ZFIN or Ensembl*	
Count	1,899	2,028	1,605	**2,283**	428
Percent (out of 2,711 human S1500+ genes)	70.0	74.8	59.2	**84.2**	15.8

The *bold* values (column “HomoloGene, ZFIN or Ensembl”) are representative of CZfO db, and were used to determine the values in the last column.

After implementing the strategy in the Materials and Methods “Building the Zf S1500+ Gene Set” section, the final Zf S1500+ gene set (Supplementary Data S4) contained 3,055 genes: 2,849 human orthologs (containing 548 genes that were also nominated by experts) and 206 additional genes from the expert-curated list. This gene set is slightly larger than human S1500+, but offers optimal coverage of the same transcriptomic space as human S1500+, and also contains some zebrafish-specific genes chosen by domain experts.

### Comparison to other S1500+ gene sets

**FIG. 3.**
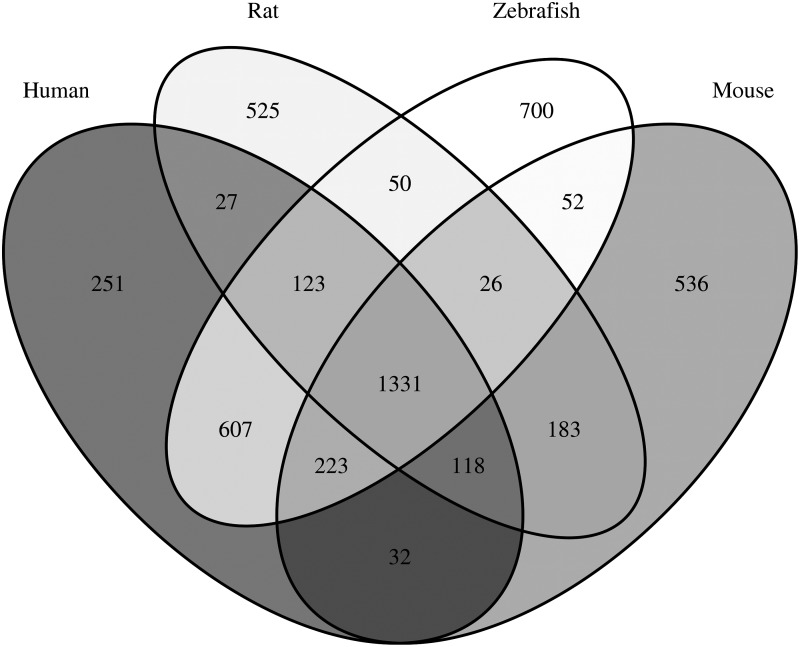
Venn Diagram of human, rat, mouse, and zebrafish S1500+ gene sets in human ortholog space. HomoloGene was used to determine human orthologs of rat and mouse S1500+ genes. CZfO db was used to determine human orthologs of zebrafish Zf S1500+ genes. CZfO db, Combined Zebrafish Ortholog Database.

Since the rat and mouse S1500+ gene sets were not predominantly developed using human orthology, we looked into the overlapping genes between all three mammalian S1500+ gene sets and Zf S1500+. Figure 3 displays the overlapping genes in human orthologous gene space (using HomoloGene to determine human orthologs of rat and mouse S1500+ genes and CZfO db for human orthologs of zebrafish Zf S1500+ genes). Therefore, species-specific genes with no human orthologs are not included in the Venn diagram. Each species' S1500+ gene set has many genes that are unique to that species (Fig. 3). However, the majority (1,331) of the genes are common between all four S1500+ gene sets. There are only 118 genes that are common among the three mammalian S1500+ set that are not in Zf S1500+.

### WTr extrapolation from Zf S1500+

The human S1500+ gene set was chosen, in part, to facilitate the extrapolation of the remaining transcriptome with high accuracy.^6^ However, there were limited transcriptomic data in zebrafish to train our extrapolation algorithm on zebrafish data directly. Therefore, we propose an alternate approach to transcriptome extrapolation from the Zf S1500+ platform by using the human extrapolation and orthologous relationships between human-zebrafish genes to achieve the goal of maximizing coverage of biological space, when analyzing Zf S1500+ expression data. This proposed approach is to begin with Zf S1500+ genes, map them to their corresponding human orthologs, and use the human orthologs to extrapolate (i.e., to all other human genes that also have zebrafish orthologs). Specifically, this “Human Extrapolation” approach would require a three-step process as follows:

(1) Map Zf S1500+ genes to their human orthologs.
(2) Extrapolate expression of orthologous human transcriptome (i.e., only those human genes with unmeasured zebrafish orthologs).
(3) Map extrapolated human genes back to zebrafish to obtain full zebrafish transcriptome (i.e., zebrafish genes without a human orthologue are excluded).

Under this approach, the resulting full zebrafish transcriptome would consist of all zebrafish genes directly measured by the Zf S1500+ gene set and those zebrafish genes extrapolated using the human-zebrafish orthologous extrapolation as described above. As a caveat to this approach, zebrafish whole-transcriptome inference was limited to a somewhat reduced transcriptome by this approach, as we will not be able to extrapolate expression of any zebrafish-specific genes that are not in the Zf S1500+ gene set.

To explore the limitations of this approach, we determined the maximum number of genes that would be possible using our existing human-zebrafish orthology mappings and our previous efforts to extrapolate human genes. A total of 25,599 unique human genes can be directly measured or extrapolated based on the human S1500+ platform, of which 18,430 are protein-coding genes. Of these, 13,728 (74.5%) human protein-coding genes have an orthologous zebrafish gene (Table 4), which is consistent with previously published homology rates for protein-coding genes between zebrafish and human.^9^

**Table 4.** Zebrafish Orthologs for Human Full (Measured+Extrapolated) Transcriptome Protein-Coding Genes

*Human full transcriptome protein-coding genes*	*Human genes with zebrafish orthologs*				*Human genes with no Zebrafish orthologs*
	*NCBI HomoloGene*	*ZFIN*	*Ensembl*	*HomoloGene, ZFIN or Ensembl*	
Count	11,340	12,346	9,204	**13,728**	4,702
Percent (out of 18,430)	61.5	67.0	49.9	**74.5**	25.5

The *bold* values (column “HomoloGene, ZFIN or Ensembl”) are representative of CZfO db, and were used to determine the values in the last column.

The subset of the 25,599 full (measured+extrapolated) human transcriptome genes that have zebrafish orthologs in CZfO db is 13,802 human genes. These human genes are orthologs of 17,118 zebrafish genes. This set of common genes (i.e., 13,802 human genes corresponding to 17,118 zebrafish genes) is the universe of an extrapolatable transcriptome using our Human Extrapolation approach. Combining this extrapolatable set with the measured Zf S1500+ zebrafish genes resulted in a total of 20,173 genes, 19,967 of which are orthologs of 13,876 human genes. This resulting set of 20,173 zebrafish genes represents the actual portion of the whole zebrafish transcriptome that can be measured or inferred using the Zf S1500+ platform and this proposed extrapolation approach. This is ∼85.5% of the of the 23,601 zebrafish GENE_INFO table genes with current Entrez and Ensembl IDs.

### Pathway coverage

The gene sets in MSigDB are a community standard for performing pathway analysis. However, the functional annotations in MSigDB are provided by only using human gene symbols, and by performing Gene Set Enrichment Analysis (GSEA). Use of any model organism data with these annotations requires mapping to human orthologs. The proposed approach can take the resulting zebrafish full (measured+extrapolated) transcriptome and use the human orthologs to perform pathway analysis. Since we are limited to human genes that have zebrafish orthologs, there may be certain biological functions that are fundamentally or mechanistically different between the two species that may result in reduced pathway coverage. We have investigated what pathway-level analyses will be feasible to assess the extent of these possible limitations based on this proposed transcriptome extrapolation approach.

We compared coverage of 1,379 C2-CP and Hallmark gene sets in MsigDB between the human S1500+ and zebrafish Zf S1500+ measured genes and between the human and zebrafish full (measured+extrapolated) transcriptomes. For human, pathway coverage was defined as the proportion of genes in a pathway that was present in S1500+ or the full transcriptome, respectively. For zebrafish, pathway coverage was defined as the proportion of (human) genes in a pathway that had an orthologous zebrafish gene present in Zf S1500+ or the full transcriptome of 20,173 genes determined above.

When the full transcriptomes were used, MSigDB pathway coverage is only ∼8.9% lower in zebrafish compared to human (Table 5). Without extrapolation, in other words, using only the measured human S1500+ or Zf S1500+ genes, MSigDB pathway coverage was comparable between zebrafish and human. Thus, the genes chosen for the Zf S1500+ platform cover a similar proportion of the biological space as represented by MSigDB pathways. However, the extent to which extrapolation can be used to expand coverage of these pathways was somewhat limited in zebrafish due to the reliance on orthology mapping. For smaller pathways, in particular, the fewer number of genes directly measured in either platform without extrapolation would not be sufficient to make sound statistical inference (e.g., GSEA). We therefore recommend extrapolation before making pathway inferences.

**Table 5.** Mean Molecular Signature Database Pathway Coverage

*C2-CP subcategory*	*Zebrafish S1500+*	*Human S1500+*	*Zebrafish full transcriptome*	*Human full transcriptome*
C2-CP	47.4	45.4	91.9	99.0
C2-CP-KEGG	38.3	36.0	85.8	98.3
C2-CP-REACTOME	39.4	37.0	86.9	95.9
C2-CP-BIOCARTA	53.6	53.0	90.8	98.0
Hallmark	40.4	38.0	88.5	98.7

Coverage (percent of measured/extrapolated genes per pathway, on average) is displayed based on the human S1500+, Zf S1500+, and human/zebrafish full extrapolated transcriptomes.

KEGG, Kyoto Encyclopedia of Genes and Genomes.

Next, we assessed whether there are specific pathways or biological functions that will not have enough gene representation based on the zebrafish full transcriptome (i.e., have fewer than 10 genes in the zebrafish-extrapolated WTr). Table 6 shows the only four pathways that can be very reliably (i.e., contain at least 20 genes) assessed using the human S1500+ platform and extrapolation, but not using the Zf S1500+ platform and extrapolation.

**Table 6.** Molecular Signature Database C2-CP Pathways with Limited Coverage in Zebrafish Extrapolated Transcriptome

*Pathway standard name*	*No. of genes in pathway*	*Zf S1500+ gene count*	*Human S1500+ gene count*	*Zebrafish full transcriptome gene count*	*Human full transcriptome gene count*
REACTOME_OLFACTORY_SIGNALING_PATHWAY	328	3	3	3	322
REACTOME_BETA_DEFENSINS	42	2	3	4	38
REACTOME_DEFENSINS	51	4	8	6	47
REACTOME_TERMINATION_OF_O_GLYCAN_BIOSYNTHESIS	24	1	3	7	20

MSigDB C2-CP pathways displayed here have at least 20 genes present after human extrapolation, but will have <10 genes in the zebrafish extrapolated WTr (after using human training data).

MSigDB, Molecular Signature Database; WTr, whole transcriptome.

REACTOME_OLFACTORY_SIGNALING_PATHWAY is a key example of a large human pathway that cannot be equivalently assessed using zebrafish genes due to a lack of known orthology. However, olfactory receptor (OR) genes are known to have higher than average sequence divergence between species compared to other gene types, and teleost fish have fewer, but more diverse (evolved from more ancestral OR genes) OR genes than mammals.^47^

Overall, the vast majority of pathways with adequate coverage after human extrapolation also have adequate coverage after zebrafish extrapolation using human training data. This olfactory pathway is the only MSigDB pathway that will have less than 5% coverage by the zebrafish full (measured+extrapolated) transcriptome. In addition, there are 243 MSigDB pathways that will have 100% coverage by the zebrafish full transcriptome (Supplementary Data S5). Thus, this proposed extrapolation approach provides sufficient coverage of pathways related to a wide range of biological functions.

### Assessment of Zf S1500+ using published whole transcriptome RNA-Seq gene expression data

To assess the utility of the genes chosen for Zf S1500+, it was necessary to test the hypothesis that WTr DEGs appeared in this gene set more frequently than in random gene sets of the same size. Then, we needed to determine whether DEGs identified by using only measured expression of the Zf S1500+ genes lead to valid pathway-level conclusions that represent the same biology that a researcher would observe when measuring the full transcriptome. In this study, we evaluated the Zf S1500+ gene set using four publicly available RNA-Seq datasets (GSE113676,^41^ GSE76564,^42^ GSE75894,^42^ and GSE100062^43^).

In addition, the extrapolation method proposed above (converting zebrafish S1500+ genes to their human orthologs, extrapolating in the human transcriptional space, and converting back to zebrafish genes), termed the “Human Extrapolation” approach, needed to be tested. The extrapolation algorithm has been extensively tested in the human transcriptomic space and benefits from the ability to train on a large dataset. However, as discussed above, this approach results in a slightly minimized zebrafish transcriptome and cannot extrapolate to zebrafish-specific genes that have no human orthologs. The only zebrafish-specific genes in the result will be the ones measured on the Zf S1500+ platform. Also, this assumes that the human intergene correlations mimic that of zebrafish and may not account for different developmental stages that zebrafish are used to study.

Ideally one would want to extrapolate in zebrafish directly to maximize the genes that can be extrapolated and to truly capture zebrafish gene interconnections and biology. This can still be done using the same statistical methods that work for human extrapolation. However, this approach, termed the “Zebrafish Extrapolation” approach, may be too limited by the smaller breadth of training data, potentially reducing accuracy. We have additionally used the four public datasets to test and compare both extrapolation methods as an extension to simply measuring and analyzing the Zf S1500+ genes.

For the BPA dataset,^41^ DEG and DEP analysis were performed comparing (1) the middle concentration, 1 ppm, to the control group and (2) the high concentration, 4 ppm, to the control group.

For the middle concentration, the Zf S1500+ gene set contained a similar number of WTr RNA-Seq DEGs to random sets of genes (Table 7 and Supplementary Data S6). The Human Extrapolation approach increased the overall number of detected DEGs, but did not increase the number of true positive (TP) DEG calls (remained 6), and analyzed a total of ∼16K genes (Table 8). Extrapolation using zebrafish training data further increased the overall number of DEGs. It also doubled the TP count (number of DEGs that overlap with the WTr DEG list) and increased the sensitivity (16.2%–21.4%) compared to the Human Extrapolation approach. This extrapolation scheme led to a total of ∼21K genes in the analysis.

**Table 7.** Count of Whole Transcriptome Differentially Expressed Genes in Zf S1500+ Gene List Compared to Random Gene Sets of the Same Size for All Gene Expression Omnibus Datasets

*Series*	*Comparison*	*Set*	*No. of genes*^a^	*No. of genes with RNA-Seq WTr data*^b^	*No. of RNA-Seq WTr DEGs in set*^c^	*Corrected percent of RNA-Seq DEGs in set*^d^
GSE113676	BPA Mid vs. Control	RNA-Seq WTr	20,239	20,239	54	
		Zf S1500+	3,055	2,983	8	14.6
		Random1	3,055	2,924	12	22.3
		Random2	3,055	2,904	6	11.2
		Random3	3,055	2,925	7	13.0
	BPA High vs. Control	RNA-Seq WTr	20,239	20,239	148	
		Zf S1500+	3,055	2,983	34	22.6
		Random1	3,055	2,924	26	17.6
		Random2	3,055	2,904	16	10.9
		Random3	3,055	2,925	21	14.2
GSE76564	Regenerating Caudal Fin vs. Uninjured	RNA-Seq WTr	17,371	17,371	371	
		Zf S1500+	3,055	2,607	79	20.6
		Random1	3,055	2,515	55	14.9
		Random2	3,055	2,484	54	14.8
		Random3	3,055	2,512	50	13.6
GSE75894	Ablated Heart vs. Uninjured	RNA-Seq WTr	17,371	17,371	545	
		Zf S1500+	3,055	2,607	107	19.0
		Random1	3,055	2,515	87	16.1
		Random2	3,055	2,484	82	15.3
		Random3	3,055	2,512	86	15.9
GSE100062	Rods vs. Nonrod Retinas	RNA-Seq WTr	17,629	17,629	1,018	
		Zf S1500+	3,055	2,619	175	16.8
		Random1	3,055	2,547	151	14.9
		Random2	3,055	2,530	128	12.7
		Random3	3,055	2,548	157	15.5

^a^ Number of genes in the set.

^b^ Number of genes in each set that were measured in the RNA-Seq dataset.

^c^ Number of DEGs from the WTr analysis of the RNA-Seq dataset that appear in each set.

^d^ Percent of DEGs from the WTr RNA-Seq dataset that appear in each set (c/b) where the count in c is corrected for the size in b with respect to the size of each subset. This is equivalent to multiplying by a correction factor of $1 + \left( { 1 - \frac { b } { { mean \;b \;for \;Zf \;S1500 + and \;random \;subsets } } } \right)$.

DEGs, differentially expressed genes.

**Table 8.** Differentially Expressed Gene Result Concordance with Whole Transcriptome Analysis for All Gene Expression Omnibus Datasets

*Series*	*Comparison*	*Set*	*No. of genes*^a^	*No. of genes with RNA-Seq WTr data*^b^	*No. of DEGs*^c^	*No. of TP DEGs*^d^	*Sensitivity (%)*^e^	*Specificity (%)*^f^	*Pearson correlation (all genes)*^g^	*Pearson correlation (extrapolated genes only)*^h^
GSE113676	BPA Mid vs. Control	RNA-Seq WTr	20,239	20,239	54	54				
		Zf S1500+	3,055	2,983	10	6				
		Human Extrapolation	16,255	15,226	32	6	16.2	99.8	0.626	0.509
		Zf Extrapolation	21,140	20,239	78	13	24.1	99.7	0.860	0.854
	BPA High vs. Control	RNA-Seq WTr	20,239	20,239	148	148				
		Zf S1500+	3,055	2,983	34	29				
		Human Extrapolation	16,255	15,226	95	31	28.2	99.6		
		Zf Extrapolation	21,140	20,239	133	59	39.9	99.7		
GSE76564	Regenerating Caudal Fin vs. Uninjured	RNA-Seq WTr	17,371	17,371	371	371				
		Zf S1500+	3,055	2,607	58	40				
		Human Extrapolation	16,479	13,554	179	46	14.8	99.2	0.667	0.571
		Zf Extrapolation	21,140	17,371	450	55	14.8	98.1	0.821	0.799
GSE75894	Ablated Heart vs. Uninjured	RNA-Seq WTr	17,371	17,371	545	545				
		Zf S1500+	3,055	2,607	119	89				
		Human Extrapolation	16,479	13,554	583	106	23.3	97.1	0.667	0.565
		Zf Extrapolation	21,140	17,371	691	141	25.9	97.4	0.847	0.828
GSE100062	Rods vs. Nonrod Retinas	RNA-Seq WTr	17,629	17,629	1,018	1,018				
		Zf S1500+	3,055	2,619	155	146				
		Human Extrapolation	16,271	13,575	806	208	25.2	96.3	0.640	0.535
		Zf Extrapolation	21,140	17,629	1,343	316	31.0	95.0	0.822	0.793

^a^ Number of genes in the set.

^b^ Number of genes in each set that were measured in the RNA-Seq dataset.

^c^ Number of DEGs for each set (determined using the thresholds described in the Materials and Methods section for each series).

^d^ Number of DEGs in c that were also DEGs in the WTr analysis of the RNA-Seq dataset. Here, TP refers to WTr RNA-Seq analysis.

^e^ Sensitivity as a percent calculated as TP/(TP + FN) × 100.

^f^ Specificity as a percent calculated as TN/(TN + FP) × 100

^g^ Pearson correlation between normalized gene expression values for the overlapping genes in WTr and specified set, calculated using all samples in the series.

^h^ Pearson correlation in g for extrapolated genes only (not Zf S1500+ genes).

TP, true positive count; FN, false negative count; TN, true negative count; FP, false positive count.

DAVID pathway analysis on the Zf S1500+ DEG list alone determined the most significant pathway from the WTr analysis (Supplementary Data S6). Random gene lists of the same size as Zf S1500+ did not lead to any pathway-level conclusions at this concentration (Table 9). The Human Extrapolation approach led to the identification of additional pathways compared to the S1500+ subset alone, which were DEPs in the WTr analysis and started to pick up on important keywords from the WTr DEPs.

**Table 9.** Differentially Enriched Pathway Result Concordance with Whole Transcriptome Analysis for All Gene Expression Omnibus Datasets

*Series*	*Comparison*	*Set*	*No. of DEPs*^a^	*No. of TP DEPs*^b^	*Percent of top 10 RNA-Seq WT TP DEPs*^c^	*Percent of RNA-Seq keywords present in DEPs*^d^
GSE113676	BPA Mid vs. Control	RNA-Seq WTr	30	30	100	100.0
		Zf S1500+	1	1	10	0.0
		Human Extrapolation	30	3	30	40.0
		Zf Extrapolation	30	23	77	100.0
		Random1	0	0	0	0.0
		Random2	0	0	0	0.0
		Random3	0	0	0	0.0
	BPA High vs. Control	RNA-Seq WTr	12	12	100	100.0
		Zf S1500+	3	3	30	62.5
		Human Extrapolation	20	6	60	100.0
		Zf Extrapolation	18	7	70	100.0
		Random1	1	0	0	0.0
		Random2	0	0	0	0.0
		Random3	0	0	0	0.0
GSE76564	Regenerating Caudal Fin vs. Uninjured	RNA-Seq WTr	30	30	100	100.0
		Zf S1500+	4	3	30	50.0
		Human Extrapolation	19	3	20	50.0
		Zf Extrapolation	21	6	30	50.0
		Random1	6	0	0	0.0
		Random2	2	0	0	0.0
		Random3	3	3	20	25.0
GSE75894	Ablated Heart vs. Uninjured	RNA-Seq WTr	36	36	100	100.0
		Zf S1500+	4	3	10	9.1
		Human Extrapolation	16	2	0	45.5
		Zf Extrapolation	23	16	70	81.8
		Random1	3	2	20	9.1
		Random2	0	0	0	0.0
		Random3	0	0	0	0.0
GSE100062	Rods vs. Non-rod Retinas	RNA-Seq WTr	78	78	100	100.0
		Zf S1500+	22	10	40	46.2
		Human Extrapolation	43	11	30	57.7
		Zf Extrapolation	71	30	50	69.2
		Random1	15	9	20	23.1
		Random2	11	2	20	34.6
		Random3	8	4	20	19.2

^a^ Number of DEPs for each set (determined using the thresholds described in the Materials and Methods section for each series).

^b^ Number of DEPs in a that were also DEPs in the WTr analysis of the RNA-Seq dataset. Here, TP refers to WTr RNA-Seq analysis.

^c^ Percent of top 10 RNA-Seq WTr DEPs (sorted by increasing *p*-value) that were among the DEPs in b.

^d^ Percent of Keywords from the WTr RNA-Seq analysis that appeared in the name of any pathway in the specified set. Keywords were determined as any one- or two-word sequence that appeared in the name of at least two WTr DEPs. Common words that did not describe useful biology (like “pathway” or “complex”) were not included as Keywords. The Keywords, frequencies, and set inclusion can be found in the Supplementary Data for each series comparison.

DEPs, differentially enriched pathways.

The Zebrafish Extrapolation approach led to many more overlapping DEPs with WTr. There were additional lipid pathways (“GO:0008289∼lipid binding” and “GO:0006869∼lipid transport”) that were determined in both extrapolated analyses that were mentioned in the accompanying article by Martínez *et al.*^41^ (based on different analysis methods that were not pairwise group comparisons), which are nonoverlapping with the RNA-Seq WTr DEP list (which did indicate “GO:0006629∼lipid metabolic process”). Although these are different pathways, they have similar biological function, and 100% (Table 9) of the biology in the WTr DEPs, as assessed by keyword matching, was identified using this extrapolation approach.

For the high BPA concentration comparison, Zf S1500+ contained more WTr DEGs (i.e., 34 compared to 16–26) than random sets of genes (Table 7 and Supplementary Data S7). The Human Extrapolation approach slightly increases the number of TPs that were detected as significant (i.e., from 29 to 31) with high specificity (99.6%), and the Zebrafish Extrapolation approach further increased the number of TPs to 59, increased the sensitivity, and modestly improved specificity compared to the Human Extrapolation approach (Table 8).

The Zf S1500+ DEG list led to the discovery of three of the top four pathways from the WTr analysis (Supplementary Data S7 and Table 9) and led to 62.5% of the keyword-level biological conclusions from the WTr DEPs. Random gene lists did not lead to any of the same biological conclusions at the pathway level. Both extrapolation approaches led to the identification of additional pathways compared to the Zf S1500+ subset alone, which were DEPs in the WTr analysis, and captured 100% of the WTr DEP keywords.

In the caudal fin dataset,^42^ genes on Zf S1500+ contained more WTr DEGs (i.e., 79 compared to 50–55) than appear in random sets of genes (Table 7 and Supplementary Data S8). Both extrapolation approaches led to a marginal increase in the number of statistically significant TP DEGs compared to Zf S1500+ (Table 8). Overall, many more DEGs, not just TPs, were determined after extrapolation, especially after extrapolation with zebrafish training data. However, sensitivity and specificity did not improve for the Zebrafish Extrapolation analysis compared with Human Extrapolation.

For this dataset, the Zf S1500+ subset determined three of the top 10 DEPs from the WTr analysis and an additional DEP in common with the Zebrafish Extrapolation analysis (Table 9 and Supplementary Data S8). The Random3 gene list determined 2 of the top 10 DEPs, while the other random gene sets did not lead to the identification of biologically relevant DEPs nor any overlap with WTr. Both extrapolation approaches led to the identification of additional pathways compared to the Zf S1500+ subset alone. Only some of these DEPs were common with the WTr analysis, but the Zebrafish Extrapolation approach did increase the types of biology that overlap to a larger degree (Supplementary Data S8).

In the ablated heart dataset, Zf S1500+ contained more of the WTr DEGs (107 compared to 82–87) compared to random gene lists (Table 7 and Supplementary Data S9). Both extrapolation approaches led to more TP DEGs than were detected in the Zf S1500+ analysis (Table 8). Overall, many more DEGs were determined after extrapolation, comparable to the number of DEGs in WTr. The Zebrafish Extrapolation approach had higher sensitivity and specificity rates than the Human Extrapolation for this dataset.

The Zf S1500+ subset discovered three of the DEPs from the WTr analysis (Table 9 and Supplementary Data S9). The Random1 gene list determined the top two DEPs, but the other randomly generated gene lists did not determine any DEPs.

The Human Extrapolation approach led to more DEPs, but not more overlap with WTr DEPs. However, there were biologically meaningful additions such as “GO:0007507∼heart development” that were missed in the other analyses. Additionally, almost half of the biology, based on WTr DEP keywords, was captured by the 16 significant pathways (Table 9). The within zebrafish extrapolation approach vastly increased the number DEPs that overlap with the WTr results (i.e., 16 TP DEPs, increased from two in Human Extrapolation, and three from Zf S1500+ genes alone). Additionally, Zebrafish Extrapolation captured 70% of the top 10 WTr DEPs and improved the ability of the results to forge biologically meaningful conclusions for this dataset.

The WTr DEGs discovered in the eye dataset^43^ appeared more in the Zf S1500+ gene lists than in the random gene sets (175 compared to 128–157; Table 7). The Human Extrapolation DEGs contained more TPs than the Zf S1500+ analysis, and the Zebrafish Extrapolation DEGs contained even more TPs (Table 8 and Supplementary Data S10). Sensitivity increased for the Zebrafish Extrapolation approach (31.0% compared to 25.2% for the Human Extrapolated method), while specificity slightly decreased.

This dataset contained many more DEGs than the others, resulting in more pathways. For this dataset, the random gene sets were able to capture some of the DEPs from WTr (2–9), but Zf S1500+ still identified more TP DEPs, a higher proportion of WTr RNA-Seq top 10 DEPs, and more of the overall biology (Table 9). All gene sets (Random1-3 and Zf S1500+) identified the KEGG Phototransduction pathway (the top WTr pathway; Supplementary Data S10). However, no random set was able to identify “GO:0007601^*^visual perception” (the third WTr DEP). This pathway was identified with the Zf S1500+ gene set. The Human Extrapolation approach improved the values for each DEP metric (Table 9), and these values continued to increase with the Zebrafish Extrapolation method.

As an additional test of the validity of extrapolated expression values coming from each approach, Pearson correlation between extrapolated and true WTr normalized gene expression values was calculated for each study for all genes in the RNA-Seq, and extrapolated datasets and for extrapolated genes only, to avoid artificially increasing the results by including the Zf S1500+ measured genes, across all samples. Based on this metric, the Zebrafish Extrapolation approach outperformed Human Extrapolation for every dataset (Zebrafish Extrapolation: 0.79–0.85; Human Extrapolation: 0.51–0.57; Table 8).

### Summary of evaluating Zf S1500+ and extrapolation using whole transcriptome RNA-Seq gene expression data

In all four datasets used to validate the Zf S1500+ gene set, more Zf S1500+ genes were measured by WTr RNA-Seq than for any of the random subsets (Table 7; column b). This, although limited, provides some evidentiary support that the genes selected for inclusion in Zf S1500+ are more transcriptionally active than and measured in more studies than random gene sets of the same size (3,055 genes). In addition, Zf S1500+ contained more genes that were differentially expressed in a wide variety of treatments/comparisons across ages and genetic backgrounds, even after correcting for the larger number of measured genes on Zf S1500+. While for some series, WTr DEGs were somewhat more prevalent in the Zf S1500+ gene set relative to the random gene sets, this corresponded to large changes in significant DEPs for all series tested.

The Zf S1500+ gene list can reach biological conclusions at the pathway level that lead to similar conclusions drawn from analysis of WTr RNA-Seq data. In some examples, a random subset of genes of the same size as the Zf S1500+ subset was able to (by chance) make valid pathway-level conclusions. However, no one random gene list performed well in all of the varied datasets tested in this study, while the Zf S1500+ subset led to some portion of the valid biological conclusions in every dataset tested. Therefore, this gene subset is a good representative transcriptomic surrogate for learning about biology of the whole organism.

Using orthology mapping and the large human RNA-Seq training data (64,514 samples) to extrapolate zebrafish genes increased the number of DEGs and DEPs compared to simply using the Zf S1500+ dataset without extrapolation. This Human Extrapolation approach did lead to making additional biologically plausible/meaningful conclusions, and in some cases, it led to more overlap with WTr RNA-Seq results.

Extrapolating within zebrafish using zebrafish training data (although limited in size: 1,791 samples) led to retaining the majority of biological conclusions from WTr RNA-Seq DEG and DEP analyses, even though the specific DEGs and DEPs did not always overlap too heavily. In addition, the Zebrafish Extrapolation approach led to a larger zebrafish whole transcriptomic space compared to Human Extrapolation, although reducing the sequencing cost compared to WTr RNA-Seq. This approach will continue to improve in accuracy as more public zebrafish RNA-Seq data become available to train these extrapolation methods.

## Discussion

The creation of a human S1500+ gene set to increase the efficiency and biological diversity of HTT, while reducing the burden of measuring the full transcriptomic space in a data-driven manner, has enabled significant progress toward the aims of Tox21.^6^ For this research, we have embarked upon a similar endeavor for the zebrafish model, which is highly used for chemical toxicity prioritization and screening.

The zebrafish model faces some difficulties in utilizing the same data-driven approach due to lack of large volume of zebrafish transcriptomics data in the public domain; therefore, we have instead created the Zf S1500+ gene set by incorporating at least one zebrafish ortholog per human S1500+ gene. This allows the resulting gene list to still benefit from the biological and pathway-level knowledge and relationships that were factored into the human S1500+ design.

While the creation of Zf S1500+ was dependent on gene-to-gene interconnectedness from human transcriptomic data, the gene set also bolsters crowd sourcing of zebrafish genes of interest to toxicologists and developmental biologists. We solicited additional gene nominations from zebrafish domain experts and incorporated such genes to create the final Zf S1500+ gene set. In doing so, we have taken steps to ensure that the directly measured genes selected to be on the Zf S1500+ platform are of high relevance to the general scientific and zebrafish-specific research communities, particularly with regard to developmental neurotoxicological research.

The zebrafish model presents an additional caveat in creating a small list of sentinel genes (to be on par with the human S1500+ gene set). Due to the whole genome duplication event in teleost fish evolutionary history,^36^ many human genes map to multiple orthologous zebrafish genes. Duplicate zebrafish genes can become inactive, take on new functions, or partition functions between gene copies. Therefore, some duplicate zebrafish genes can be mechanistically different and may be of unique interest to researchers studying specific biological functions.

A sentinel gene set seeks to measure a transcriptomic subset that can be used to gain full transcriptome knowledge. To keep the size of the Zf S1500+ gene set manageable for eventual HTT platform assay creation and similar in size to the human S1500+, we have used an approach that removes many of the redundant duplicate gene copies from the final ∼3K gene set. However, by keeping all nominated genes with noted biological function, we hope that distinct gene copies of importance to the zebrafish community have been retained in the proposed Zf S1500+ list. Although signal can be extrapolated to the majority of nonmeasured genes, researchers are welcome to measure additional genes of interest to their research group when utilizing this gene set.

We have evaluated the genes on Zf S1500+ for their ability to lead researchers to the same biological conclusions that would have been drawn if they measured expression of the WTr. We have done this by showing the DEG and DEP overlap between an analysis performed using only the Zf S1500+ genes and an analysis using full publicly available RNA-Seq datasets. We have demonstrated that Zf S1500+ genes alone can capture a large portion of the biology similar to WTr analysis in multiple datasets. Reliance on zebrafish orthologs of human S1500+ genes and expert nominations to determine sentinel zebrafish genes did lead to a biologically meaningful zebrafish gene set. We have also shown that this set captures relevant biology in varied datasets unlike random gene sets of the same size.

As the available breadth of zebrafish transcriptomic data continues to grow, the ability to assess this gene set for zebrafish-specific research will increase. For example, toxicant gene expression data may cause fewer DEGs relative to tissue ablation, so inclusion of varied types of studies in the training dataset is important. An additional caveat may be that many zebrafish toxicology RNA-Seq experiments use whole embryos and larvae, which dilutes read counts and makes it more difficult to detect xenobiotic-induced DEGs.

The Zf S1500+ gene set is immediately available to use in any HTT platform, and a TempO-Seq platform (BioSpyder Technologies, Inc.) is currently being developed with probes to measure all genes in the gene set, as has been done with the human, rat, and mouse S1500+ gene sets. We expect that Zf S1500+ will be a valuable subset of genes to measure in a variety of applications.

For example, the human S1500+ gene set has been used to identify genes that are differentially expressed in induced pluripotent stem cell-derived hepatocytes exposed to 21 petroleum substances compared to dimethyl sulfoxide (DMSO) controls, leading to a transcriptional toxicity profile clustering of these complex substances.^48^ The human S1500+ TempO-Seq platform has also been coupled with concentration–response modeling approaches to characterize and interpret hepatic responses to chemical exposures.^7^

The rat S1500+ TempO-Seq platform has been validated against other sequencing technologies, allowing the gene set to be used for analysis of differential gene expression in rat livers exposed to chemicals from five different modes of action.^8^ Measuring expression of the Zf S1500+ gene set using any HTT platform will enable similar high-throughput chemical or other screening approaches in zebrafish, a whole organism ideal for large-scale chemical screens, at a lower cost.

By relying first on genes selected to maximally cover the human biological response space, favoring genes representative of unique co-expression (co-regulated) clusters, while maximizing pathway coverage,^6^ we have created a zebrafish platform that benefits from the vast transcriptomic data available in the human domain. This ensures that the measured genes from this platform can be used to adequately extrapolate expression values for the whole organism. Extrapolation can capture 85.5% of the zebrafish WTr through conversion to human orthologs. We have assessed this extrapolated representation of the zebrafish transcriptome for pathway-level impact by looking into MSigDB canonical and hallmark pathway gene coverage and have shown the extrapolatable zebrafish transcriptome to adequately cover the majority of the same pathways that are covered by human S1500+ extrapolatable genes.

Researchers can use this gene set as a starting point to extrapolate whole organism transcriptional signal in zebrafish. We have discussed two proposed whole-genome extrapolation methods: (1) using human orthologs of the Zf S1500+ to extrapolate in human transcriptomic space and converting genes back to zebrafish identifiers or (2) extrapolating directly in zebrafish using a much smaller training set of zebrafish RNA-Seq gene expression data. The first approach relies on biological interconnectedness and gene-gene correlations from human transcriptomic data. To that end, this approach will not be able to extrapolate expression signal for any zebrafish genes not measured in the Zf S1500+ gene set, which do not have human orthologs. However, due to the inclusion of expert-recommended zebrafish genes, many zebrafish-specific genes covering a wide range of biological function are directly measured in the set.

We have tested both extrapolation procedures using the subset of Zf S1500+ genes from four publicly available RNA-Seq datasets that are not in the zebrafish training set. We have found that both methods provide biologically meaningful results that increase the knowledge gained from analysis of Zf S1500+ genes alone, without the added cost of measuring the WTr, and align well with results that would have been obtained by measuring expression of the WTr. For example, the Human Extrapolation approach can identify multiple fin-related pathways in a fin regeneration experiment, even though humans do not have fins. However, as one would predict, extrapolating within zebrafish is more accurate and leads to more overlapping DEG and DEP calls with the WTr analysis in some datasets.

As the breadth and diversity of public transcriptomic data for zebrafish continue to grow, performance for extrapolation of zebrafish S1500+ expression data based on zebrafish RNA-Seq training data can continue to improve, allowing for WTr extrapolation methods using zebrafish Zf S1500+ data similar to that used for the analysis of data generated from human, rat, and mouse S1500+ platforms.^49^ This evolving improvement of zebrafish extrapolation methods will allow researchers to utilize the Zf S1500+ gene set to measure the ∼3K zebrafish genes and extrapolate to a total of more than 20K genes, representing the whole zebrafish transcriptome for analysis of pathway perturbations.

The S1500+ sentinel gene set for human, rat, and mouse boast the promise of four critical objectives: (1) capturing maximal variation in gene expression, (2) being extrapolatable to reduce gene set size, (3) providing maximal, robust biological pathway coverage, and (4) containing genes particularly relevant for toxicity and disease evaluations.^5^ As the use of these sentinel gene sets continue to grow, the addition of a zebrafish gene set will be especially useful in HTT screens due to their rapid and *ex vivo* development, and the many phenotypes that are easily observed in transparent embryos and larvae. Zebrafish are an ideal model organism for assessing development and xenobiotic toxicity.^50^

The Zf S1500+ gene set includes expert-nominated genes spanning a wide range of biological processes and many genes expressed during development of a variety of biological systems (Table 2), in addition to zebrafish orthologs of all human S1500+ genes (for which orthologs exist). Zf S1500+ will be useful for gaining mechanistic understanding in a whole organism that lends itself naturally to high-throughput study designs, and it will additionally aid in making inferences about toxicogenomic outcomes for toxicologists, biologists, and geneticists alike.

## Supplementary Material

Supplemental data

Supplemental data

Supplemental data

Supplemental data

Supplemental data

Supplemental data

Supplemental data

Supplemental data

Supplemental data

Supplemental data
